# Supplementary material for: Identification of Cross Talk between FoxM1 and RASSF1A as a Therapeutic Target of Colon Cancer
Source: Cancers (Basel). 2019 Feb 8;11(2):199. doi: 10.3390/cancers11020199 (PMC6406751; doi:10.3390/cancers11020199)
Supplement: Supplementary file 1 [file cancers-11-00199-s001.pdf]

cancers

MDPI

Supplementary Materials

Identification of Cross Talk between FoxM1 and RASSF1A as a Therapeutic Target of Colon Cancer

Thomas G. Blanchard, Steven J. Czinn, Vivekjyoti Banerjee, Neha Sharda, Andrea C. Bafford, Fahad Mubariz, Dennis Morozov, Antonino Passaniti, Hafiz Ahmed and Aditi Banerjee

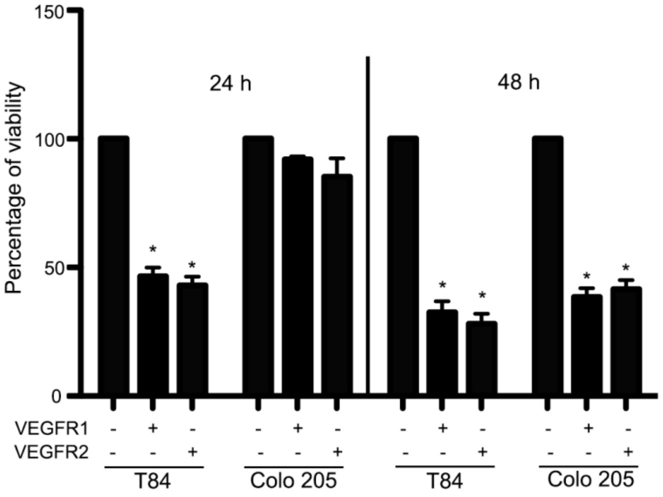

Figure S1. Neutralization level of VEGFR1 and VEGFR2.

Table S1. qRT-PCR Primers.

Gene	Primer Sequence Forward	Primer Sequence Reverse
MXI1	5′-GGAAAAGAATCGACGAGCTCAT-3′	5′-GGGTGCAGTCTGGTCCTAGTG-3′
POLR2A	5′-GCATGGCAGAGGAGTTTCGGCT-3′	5′-ATTTCCCCGGGATGCGCAATGG-3′
E2F6	5′-AGAGCTTTTCTCATGGACACA-3′	5′-CGAGACCCCATCAACGTG-3′
EZH2	5′-GCCAGACTGGGAAGAAATCTG-3′	5′-TGTGCTGGAAAATCCAAGTCA-3′
ZNF687	5′-ATGTCTGTCCTGAGTGTGGG-3′	5′-TTGTGGAAAACCTCGCAGTG-3′
NR2F1	5′-CAGCGGTGCAAACCATGTGC-3′	5′-GCAAGTTGGCTGAACCAAATG-3′
MNT	5′-GGTGGCCCTTGTTTACAGTG-3′	5′-TGGAGCATGAACAGCCACTA-3′
GABPA	5′-TTGGCAAGTCAAGAACAACAGA-3′	5′-GCGCTCTTTGTACTTTGGCT-3′
TARDBP	5′-GCTGATGGGCTGAGAACATG-3′	5′-GGACACAGAACTGCAGCAAA-3′
ZFX	5′-GGCAGTCCACAGCAAGAAC-3′	5′-TTGGTATCCGAGAAAGTCAGAAG-3′
REST	5′-AGCTGCTGTGATTACCTGGT-3′	5′-ACAGGCTGAGGTTCTACGAC-3′
ZNF592	5′-ACAGCCATCCTAGCAACAGT-3′	5′-AGAGATCCTTTGTGTGGCCA-3′
